# Mechanism of fibroblast growth factor 21 in cardiac remodeling

**DOI:** 10.3389/fcvm.2023.1202730

**Published:** 2023-06-21

**Authors:** Zeyu Zhao, Xuemei Cui, Zhangping Liao

**Affiliations:** ^1^ Queen Mary College, Nanchang University, Nanchang, China; ^2^ Fourth Clinical Medical College, Nanchang University, Nanchang, China; ^3^ Jiangxi Provincial Key Laboratory of Basic Pharmacology School of Pharmaceutical Science, Nanchang University, Nanchang, China

**Keywords:** fibroblast growth factor 21 (FGF21), heart remodeling, energy metabolism, oxidation, inflammation, autophagy

## Abstract

Cardiac remodeling is a basic pathological process that enables the progression of multiple cardiac diseases to heart failure. Fibroblast growth factor 21 is considered a regulator in maintaining energy homeostasis and shows a positive role in preventing damage caused by cardiac diseases. This review mainly summarizes the effects and related mechanisms of fibroblast growth factor 21 on pathological processes associated with cardiac remodeling, based on a variety of cells of myocardial tissue. The possibility of Fibroblast growth factor 21 as a promising treatment for the cardiac remodeling process will also be discussed.

## Introduction

Cardiac remodeling refers to a series of changes in the heart that lead to increased ventricular dilation, cardiac dysfunction, and molecular changes which can be caused by a diminished variety of factors (1), such as myocardial infarction (MI), hypertension, obesity, and valvular heart diseases (2). Numerous kinds of cells, including endothelial cells, immune cells, and fibroblasts, are involved in the remodeling process, which aims to cope with risk factors, reduce myocardial damage and initiate repair processes to fill the damaged area (3). However, cardiac remodeling can also lead to diminishing cardiac contractility and a restricted supply of energy substrates and oxygen, resulting in elevated levels of cardiac fibrosis and hypertrophy (4, 5). Although cardiac remodeling is initially considered an adaptation to restore cardiac function after damage. The unlimited pathological change results in cardiomyocyte disarrangement and dysfunction (6), eventually leading to heart failure (HF), the outcome of cardiac adverse events (7). Accordingly, the use of interventions in the early stages to preserve the structure and cardiac function is necessary to delay the progression and improve patients' quality of life (8).

One potential intervention for cardiac remodeling is the use of fibroblast growth factor 21 (FGF21). FGF21 is initially identified in the liver and subsequently is also found to be expressed in brown adipose tissue, skeletal muscle, and pancreas, where it shows its effects mainly through a paracrine manner (9). While the heart is traditionally viewed as an effector organ of FGF21, studies have also shown that it can produce FGF21 in response to damage. By binding to the receptor fibroblast growth factor receptor (FGFR)1 and cofactors *β*-klotho, FGF21 activated the downstream genes to play a crucial role in cardioprotection (10). However, a recent study has pointed out that FGF21-FGFR4 signaling promoted cardiac hypertrophy (11).

After damage occurs, cardiomyocytes secrete FGF21 in an autocrine manner and reduced myocardial injury (12). Besides, FGF21 originating from other tissues has been shown to contribute to the limitation of cardiac damage and the reversal of cardiac remodeling. Following MI, the liver played a significant role as a major source of circulating FGF21 levels (13). Additionally, FGF21 derived from brown adipose tissue was found to attenuate adverse cardiac remodeling in mice with hypertension (14). Studies have shown that FGF21 resists cardiac remodeling and the following damage through ways such as promoting autophagy, alleviating inflammation, oxidative stress, and regulating energy metabolism (12, 15–17).

FGF21 has been shown to play a protective role in various cardiovascular diseases including MI, atherosclerosis, and diabetic cardiomyopathy in different studies (18–20), and FGF21 knockout mice tend to present pathological phenotypes (21). Moreover, FGF21 levels predict the prognosis of many heart diseases such as hypertension, dilated cardiomyopathy, and MI (22–24).

**Figure 1:**
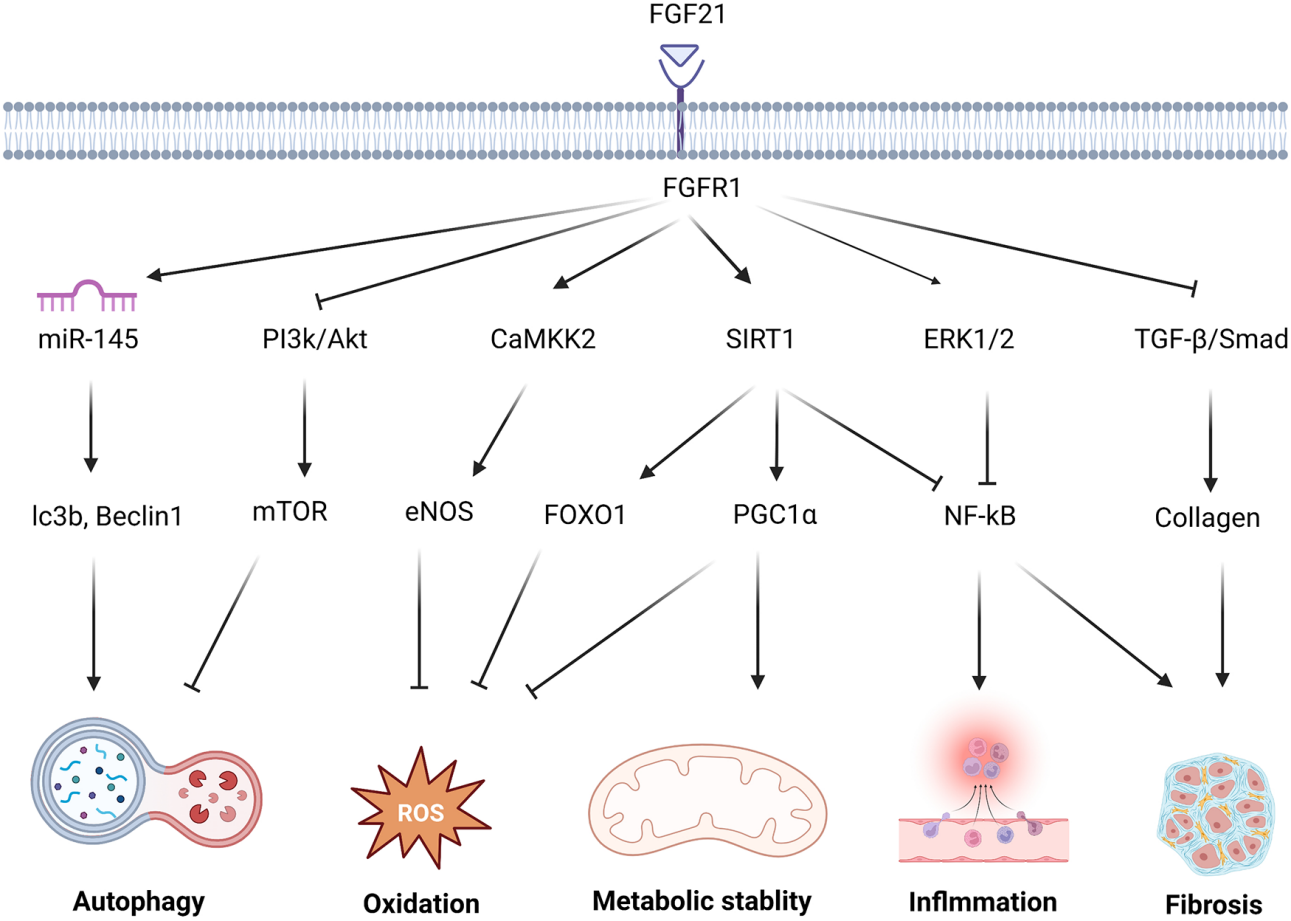
The molecular mechanism and effect of FGF21 in protecting against cardiac remodeling. FGF21 regulates multiple signaling pathways to protect against cardiac remodeling. FGF21 inhibited PI3K/Akt/mTOR pathway and upgraded the miR-145 level to enhance the autophagy level. FGF21 promotes CAMKK2-eNOS to elevate the vasomotor capacity and alleviate oxidative stress injury. via activation of FOXO1 and PGC1 *α*, SIRT1 results in multiple antioxidant proteins and transcription factors associated with oxidative stress levels. NF-kB, as a key transcription factor related to inflammation, under the action of FGF21, is inhibited by SIRT1, ERK1/2. FGF21 attenuates this adverse alteration. Reducing TGF-*β*/Smad signaling also helps avoid the secretion of large amounts of collagen by fibroblasts which may cause collagen deposition and fibrosis.

Recent studies report that FGF21 is involved in the prevention of cardiac damage. The review examines the mechanisms of FGF21 against cardiac remodeling, providing potential survival benefits through a range of pathways including autophagy, metabolic stability, oxidation, inflammation, and fibrosis. The mechanism by which FGF21 exerts protective effects on the heart are listed in Figure 1. Additionally, this review discusses the potential implications for clinical research on FGF21-related drugs.

## FGF21 in autophagy

Cellular autophagy is a process that makes cellular energy reuse by degrading autophagic vesicles containing cytoplasmic material. It is a positive implication for cardiomyocytes in resisting stress (25, 26). Recycling misfolded proteins back as energy substrates reduces the demand of cells for external nutrients, removing mitochondria residuals, toxic metabolic substrates, and reactive oxygen species (ROS) in mitochondrial dysfunction (27).

The activation of autophagy helps to maintain cardiac homeostasis and prevent myocardium hypertrophy. The energy provided by autophagy supports cell survival, helping to maintain the number of cardiomyocytes and preserve cardiac function after stress (28). A study has reported that after MI, autophagy limited cardiac damage by reducing the scar size and holding cardiac function (29). The inhibition of autophagy would lead to pronounced signs of cellular hypertrophy in the myocardium (28). Angiogenesis is beneficial for improving postinfarction collateral perfusion, reducing infarct size, and attenuating the negative effect on contractility of the infarcted area, and autophagy plays a role in initiating angiogenesis (30).

Both mTORC1 and Beclin1 are autophagy-associated molecules, playing key roles in cardiac plasticity (31). As a serine/threonine kinase, mTORC1 acts to inhibit autophagy activity at various levels. By inhibiting the transport of nuclear transcription factor EB(TFEB), mTORC1 reduced the transcription of autophagy related (Atg) genes (32). The phosphorylation of DCP2 induced by mTORC1 made specific Atg mRNA degradation (33). The autophagosome formation discontinued after the phosphorylation of unc-51-like autophagy activating kinase 1 (Ulk1) at Ser757 (34). Beclin1 is another well-known protein in autophagy initiating, regarded as a marker of autophagy activity. By protein interaction, Beclin1 was one of the vital components of the Class III phosphatidylinositol 3-kinase (PI3K)-III complex, which acted as a platform to recruit multiple proteins to form mature autophagosomes (35).

FGF21 in cellular autophagy can be beneficial for attenuating cardiomyocyte damage after stress. As a peroxisome-proliferator-activated receptor agonist, the protective effect of Fenofibrate in the diabetes-induced cardiac remodeling was depended on FGF21- Sirtuin-1 (SIRT1) activated autophagy (17). The lack of FGF21 led to the impairment of autophagy and accumulation of fatty acid, making high-fat-diet mice cardiomyocytes hypertrophy (36).

FGF21 regulated autophagy through different pathways. By increasing TFEB transcriptional activity, FGF21 administration enhanced the autophagy effect and relieved the ischemia-reperfusion (I/R) injury in vascular endothelial cells (37). FGF21 protected against I/R injury, enhancing cell autophagy and reducing inflammation through regulating miR-145, with the upregulation of lc3b (lc3b I/II) and Beclin1 (38). Through KEGG pathway analysis, the PI3K-Akt-mTOR pathway was thought to be associated with FGF21. FGF21 promoted autophagy by inhibiting PI3K-Akt-mTOR transduction (39). Another study proved that FGF21 inhibited atherogenesis by up-regulating autophagy, enabling cholesterol efflux and reducing lipid accumulation in foam cells, this effect was related to the activated rack-1 pathway (18).

## FGF21 in energy metabolism

The alteration in energy supply is closely associated with pathological cardiac hypertrophy. They can even precede the appearance of a detectable cardiac hypertrophic result. The shift of substrate metabolism was viewed as an important feature of cardiac hypertrophy (40). Physiologically, cardiac energy was supplied by fatty acids, and approximately 70% ATP was provided by lipid substrates (41). However, cardiac tissue still preserved the ability to use various types of energy substrates including ketone bodies under different conditions, which was known as metabolic flexibility (42). Since deviating in metabolic pathways simultaneously led to cardiac hypofunction and maladaptive hypertrophy, the inhibition of related pathways might restore regular metabolic capacity while attenuating cardiac remodeling.

Cardiac diseases can exhibit diverse metabolic preference profiles. Heart failure relied on glycolysis and lactate for energy, whereas diabetes-induced cardiomyopathy was typically characterized by a dominance of lipid metabolism (43–45). For late-stage patients, the instability of the energy supply directly affected contractile function, and the limited metabolic pathways further interfered with ATP generation, ultimately causing HF (40, 46). Based on a proteomic study, FGF21 was found to restore the levels of pyruvate kinase isozymes M1/M2, which improved the energy supply after I/R injury (47).

FGF21 acted on upstream signals to regulate AMPK(Adenosine 5′-monophosphate -activated protein kinase)/SIRT1/PGC(peroxisome proliferators-activated receptor *γ* coactivator)-1*α* pathways. AMPK/SIRT1/PGC-1a was considered a target pathway involved in the regulation of metabolic stability, with its effects including the coordination of ATP levels, modulation of mitochondria function, and lipid metabolism (48–51). CD36 was a fatty acid translocase that can activate the AMPK pathway to accelerate lipid metabolism under fatty acid mediation (52). In FGF21 knockout mice's hearts, there was severe lipid accumulation and CD36 levels upregulation, accompanied by a decrease in phosphorylation levels of AMPK protein and PGC-1*α* expression, showing that CD36 cannot activate AMPK/SIRT1/PGC-1*α* pathway without FGF21 presence and thus led to metabolic dysregulation (16, 53). LKB1 was essential for AMPK activation. The inhibition of LKB1 weakened FGF21 promoting effect of mitochondrial function, demonstrating that the benefits of FGF21 promoting mitochondrial synthesis and mitochondrial oxidative capacity required the AMPK/SIRT1/PGC-1*α* pathways (54).

A study using angiopoietin 2-induced cardiac hypertrophy mice noted that FGF21 treatment increased SIRT1 deacetylation activity, promoted AMPK phosphorylation, and reduced cardiac hypertrophy in a SIRT1-dependent pathway (55). In brown adipose tissue, upon adenosine binding to the surface receptor A_2_AR, released FGF21 can be upregulated by phosphorylating AMPK and PGC-1 *α* against cardiac hypertrophy (14). In MI model mice with the activated AMPK/SIRT1/PGC-1*α* pathway, circulating FGF21 levels were greatly higher even at the early onset and lasted up to a week (56). The ability of the FGF21-AMPK pathway to rapidly respond to early ischemic injury suggested that it may serve as a key to reducing MI injury.

## FGF21 in oxidation

ROS-mediated oxidative stress injury contributed to the progression of cardiac remodeling and HF. The balance between various oxidant and antioxidant enzymes, including catalases, glutathione peroxidases, xanthine oxidases, and NADPH oxidase (NOX), generally maintains ROS levels at low levels. In situations of overloading or insufficient blood supply, mitochondrial dysfunction made hearts generate insufficient ATP, increased oxidative stress (57), overproduction of hydrogen peroxide, which ultimately led to cardiomyocyte necrosis, left ventricular dysfunction, and eventually HF (58). The experiment proved that cardiomyocyte apoptosis induced by oxidative stress was one of the reasons for myocardial decompensation (59).

ROS promoted the imbalanced growth of cardiomyocytes through multiple pathways, ultimately causing cardiac hypertrophy and remodeling (60). By directly activating the transcription factor NF-kB, ROS induced the transcription of more pro-hypertrophic genes (61). NADPH oxidase was a major source of superoxide in the cardiovascular system (62). The reaction between excessive superoxide anion and Nitric Oxide (NO) formed the peroxynitrite (ONOO-), decreasing NO bioavailability, causing vasoconstriction, and leading to the early formation of atherosclerotic plaques (63). Animal studies have shown a positive relationship between increased levels of oxidative stress and disease progression in multiple animal models of HF (64–66). Similarly, FGF21 levels were also specifically elevated in patients with poor prognosis HF (67), a coincidence that has led to thoughts about a possible relationship between FGF21 and the effects of oxidative stress.

In cardiomyocytes, FGF21 production was regulated by ROS level in an autocrine manner. High levels of ROS, which were stimulated by calcium ions released from endoplasmic reticulum, triggered a signal transduction pathway known as the unfolding protein response (UPR) which activated several transmembrane sensors, ultimately resulting in elevated FGF21 expression levels (68).

FGF21 treatment improved resistance to oxidative stress by elevating the expression levels of oxidative enzymes such as superoxide dismutase (SOD) and glutathione (69). It also activates nitric oxide synthase (eNOS) to increase NO production and reduce oxidation, guaranteeing the normal contractile function of blood vessels and reducing the adverse effects of peroxides on cells (70).

Studies have shown that FGF21 induced the expression of antioxidant genes. Through the Sirt1-FOXO1 pathway, FGF21 enhanced catalases, SOD2 levels, and the pro-apoptotic protein BIM lessened (55). In the absence of FGF21, SIRT1 effects that induced the levels of uncoupling protein (UCP)3, peroxiredoxin5, glutathione peroxidase1, catalase, and sequestosome1 were abolished (71).

Another study illustrated that recombinant FGF21 activated CaMKK2/AMPK*α* signaling, enhancing eNOS phosphorylation and reducing oxidative stress responses to ameliorate diabetes-induced aortic endothelial disorders (72).

Via AMPK/PGC1*α*/SIRT1 signaling, FGF21 led to a rise of nuclear factor erythroid 2–related factor 2 (Nrf2) and downstream antioxidative enzymes activity such as NOX4, UCP2 (73).

For atrial remodeling induced by oxidative stress, FGF21 functioned by regulating the degradation of myofibrils and levels of calpain, a calcium-dependent protease, which helped to maintain cardiac function, improved arrhythmia symptoms, and preserved electrophysiological function (74).

## FGF21 in inflammation

The main reason for acute inflammatory responses is cardiomyocyte necrosis in response to various adverse factors. After damage happened, innate immune cells localized in the heart were activated, recognizing endogenous damage-associated molecular patterns, clearing necrotic cells, and releasing proinflammatory factors. Circulating immune cells were then recruited, further infiltrating the damaged area (75). This early inflammatory response was necessary to initiate the repair process, protecting the heart from further damage. However, an uncontrolled chronic inflammatory response can further aggravate cardiac adaption (76).

Chronic inflammation was one of the most important stages in the progression process of cardiac remodeling. Patients with HF have shown significantly elevated levels of proinflammatory cells and chemokines. Inhibition a series of pro-inflammatory chemokines including IL-1 and TNF-α was beneficial for attenuating adverse cardiac remodeling, maintaining cardiac function, and reducing fibrotic area (77, 78).

Proinflammatory cell activation potentiated inflammatory responses through different pathways, and their phenotypic heterogeneity dictated that they played distinct roles in cardiac inflammation.

Despite comprising only 6%–8% of noncardiomyocytes in normal cardiac tissue, cardiac macrophages massively migrated and proliferated in the remote area during the chronic healing process of MI to mediate the onset of distant myocardial remodeling, and high levels of blood-derived macrophages had a strong link to heart failure with reduced ejection fraction (HFrEF), whereas heart confined macrophages might be more involved in post-MI repair (79).

Plasma FGF21 levels were significantly elevated in patients with HFrEF, regardless of the presence of cardiac cachexia. This elevation was independently associated with the inflammatory marker IL-6, suggesting that the inflammatory response was a contributing factor to elevated FGF21 levels (80).

Among peripheral blood leukocytes, FGF21 was highly expressed in monocytes and neutrophils (81). By increasing the expression of GLUT-1, FGF21 upregulated the ability to uptake glucose in activated monocytes, which can be beneficial in immune activation (82). FGF21 played a crucial role in aiding in clearing damaged cell remnants in phagosomes, resulting from the upregulation of NOX2 transcription and expression (81).

In addition to reducing infiltration of circulating immune cells, FGF21 shifted innate macrophages towards the anti-inflammatory M2 subtype rather than pro-inflammatory (83). FGF21 negatively regulated the early formation of atherosclerotic plaques by preventing the transformation of macrophages into foam cells and reducing macrophage migration by inhibiting the NF-kB signaling pathway (84).

CD4 + Th17 cells were thought to be directly involved in the progression of adverse remodeling from hypertrophy to HF in the situation of pressure overload (85). FGF21 regulated STAT3/ROR*γ* phosphorylation and pro-T-cell expansion IL-23 levels, reducing splenic Th17 cell differentiation and proliferation, and ultimately downregulating the levels of pro-inflammatory factor IL-17, thereby alleviating arthritis in mice (86). This suggests that FGF21 can induce inflammation by downregulating T cells through the regulation of the Th17-IL-17 axis.

Multiple studies have demonstrated hemagglutination propensity, platelet activation, and levels of associated adhesion molecules all increase in HF patients (87). After activation and adhesion to the capillary wall, platelets secreted a variety of pro-inflammatory factors, promoted macrophage infiltration, and regulated precursor cell to foam cell transformation, ultimately leading to small vessel endothelial thickening and sclerotic plaque formation of large vessels. Agglutination of platelets following an acute cardiac event further enhanced microvascular obstruction and potentiated the inflammatory response (88).

To reduce reperfusion injury after MI, antiplatelet and antithrombotic drugs are routinely used in the clinic (89). FGF21 reduced factor VII expression which was a hallmark of the extrinsic coagulation pathway. Variations in the fluorescence intensity levels of platelet surface markers proved that FGF21 inhibited platelet activation, reducing hemagglutination. By regulating the ERK1/2 and TGF(Transforming Growth Factor)-β/Smad2 pathway, FGF21 promoted the expression of tPA and suppressed the expression of PAI, so that fibrinolysis was activated without bleeding risk (90).

**Figure 2:**
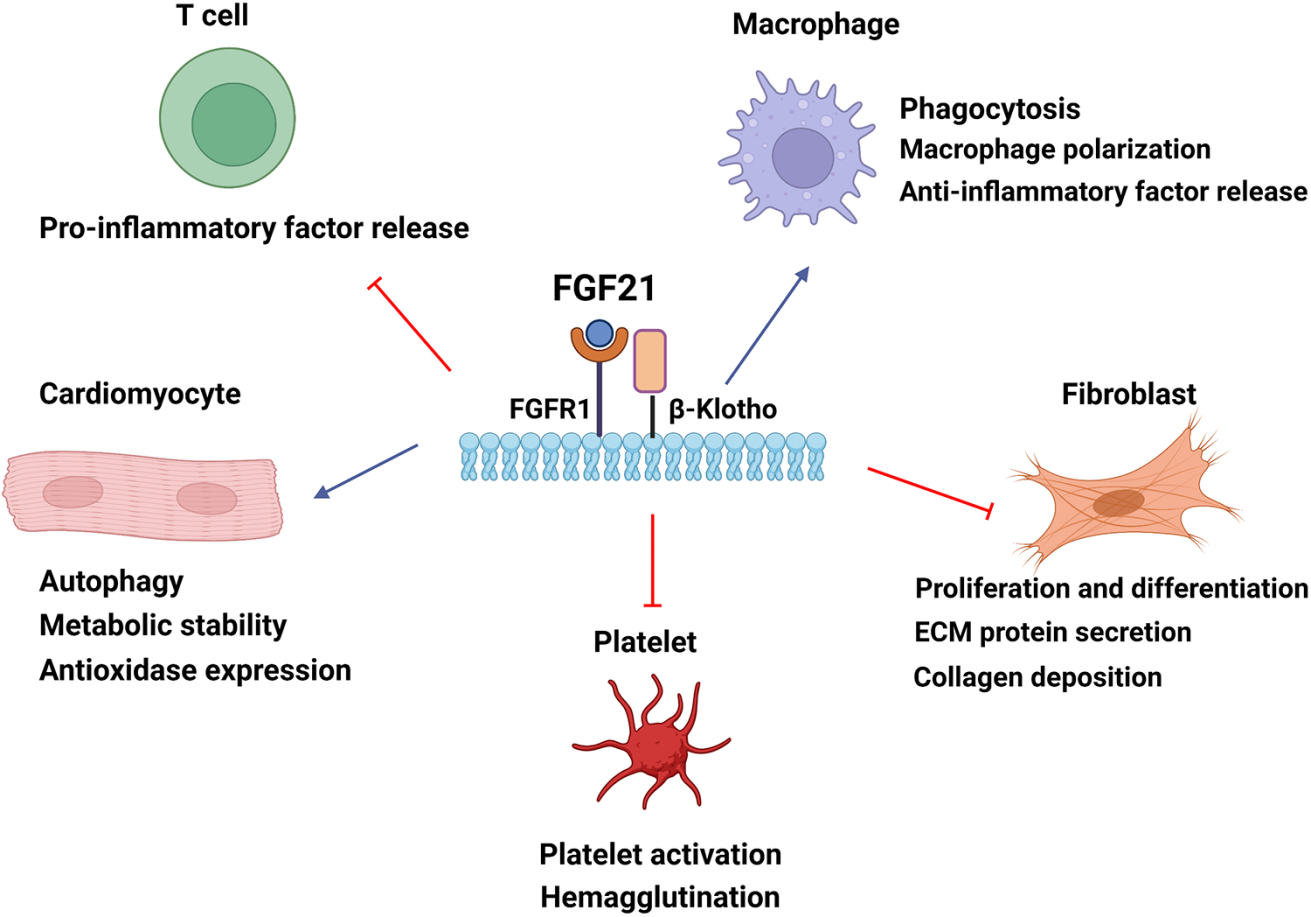
The cellular mechanisms by which FGF21 confers cardioprotection in cardiac tissue. FGF21 modulates cardiomyocyte energy metabolism and autophagy and improves oxidative stress damage. FGF21 can mediate macrophage polarization to the anti-inflammatory subtype and regulate cytokine levels to alleviate inflammatory responses. FGF21 upregulates phagocytosis to eliminate damaged remnants. The inhibition of fibroblast reduces collagen deposition of the extracellular matrix and ameliorates cardiac fibrosis by FGF21. FGF21 also downregulates hemagglutination by reducing platelet activation. Blue arrow, upregulation; red arrow, downregulation. ECM, extracellular matrix.

FGF21 presented anti-inflammatory properties by modulating the balance between pro-inflammation and anti-inflammation factors. The administration of FGF21 led to a downregulation of several pro-inflammatory factors and increased the expression of anti-inflammatory cytokines over a long period (83). Another *in vitro* experiment pointed out that this kind of action of FGF21 was related to IL-10 in the ERK1/2 pathway (15). Additionally, FGF21 alleviated inflammation in vascular endothelial cells through SIRT1-mediated NF-kB deacetylation (91). Figure 2 summarizes the roles of FGF21 in different cells of myocardial tissue.

## FGF21 in cardiac fibrosis

Fibrosis is considered as a crucial process in heart remodeling. After encountering pressure, fibroblasts secreted a large number of collagen fibers to the interstitium, and this excessive fiber deposition triggered the disarrangement of cardiomyocytes (92). The hypertension phenotype presented an increased wall/lumen ratio in resistance arteries, which was related to the activation and decomposition of collagens and other extracellular matrix components (ECM) components (93). After MI, fibroblast activation resulted in the deposition of collagen to form a scar, which helped to ensure the intactness of residual myocardial tissue and avoided serious consequences such as cardiac rupture (4, 94). During the post-infarction repair period, the distal area of the myocardium without infarction had massive fibrin deposition, leading to interstitial remodeling, reducing cardiac compliance, and promoting ventricular diastolic dysfunction development (95).

By using an efficient carrier, FGF21 administration attenuated cardiac fibrosis, resulting in decreased mRNA levels of cardiac hypertrophy and fibrosis markers in diabetic cardiomyopathy mice (96). FGF21 released from brown adipose tissue ameliorated cardiac fibrosis and hypertension-induced myocardial remodeling (14). In vitro experiments showed that FGF21 significantly decreased myofibroblast markers *α*-SMA and ACTA2 mRNA levels, suggesting that FGF21 was related to attenuating cardiac fibrosis (14, 97). The administration of the homolog of FGF21, LY2405319 in mice with liver fibrosis, reduced the levels of type 1 collagen and *α*-SMA in tissue, resulting from inhibiting the succinate- G-protein coupled receptor 91 pathway (98).

TGF*β* is one of the key factors leading to cardiac remodeling, accelerating fibroblast proliferation, and inducing accumulation of ECM components, which led to impaired cardiac function (99). FGF21 treatment attenuated the fibrotic phenotype by upregulating the transcription factor EGR1 (100). FGF21 also decreased collagen synthesis in MI mice through TGF-*β*1/Smad2/3-MMP (metalloproteinase)2/9 signaling (101). FGF21 downregulated MMP9 levels through NF-kB [Z.-C (102)]. Through the FGFR1/Syk/NLRP3 inflammasome pathway, FGF21 restrained the proliferation and migration of vascular smooth muscle cells and neointimal hyperplasia, marked by increasing in PCNA, cyclin D1 and MMP9 (103).

Galectin(Gal)-3 is mainly secreted by macrophages in the heart, released into the cytoplasmic matrix in association with collagen receptor action. It can promote macrophage migration, cardiac fibroblast proliferation, and collagen deposition in the matrix, leading to reduced ejection fraction (104). FGF21 regulated Gal-3 levels in H9c2 cells in a dose-dependent manner and ECM-related proteins (fibronectin, collagen I) levels were also decreased under FGF21 treatment (105). This suggested that FGF21 may play a protective role in the heart by reducing the level of cardiac fibrosis through downregulating Gal-3 levels. However, due to the inadequacy of the available evidence, more studies are still needed to further determine the relationship between FGF21 and Gal-3.

## Clinical application and prospects

FGF21, as a metabolically associated protective factor, has been shown to have potential clinical applications in various diseases. Elevated serum levels of FGF21 have been observed in response to stress stimuli and have been associated with a higher risk of morbidity and poor prognosis in multiple diseases. One analysis indicated that FGF21 levels were useful in predicting the severity and prognostic risk in patients with community-acquired pneumonia (106). Clinical studies have also shown that FGF21 is useful in predicting HF (107–110). Serum FGF21 levels were higher under acute insufficient sleep conditions when adipose tissue FGF21 promoter region was methylated (111).

Due to the short half-life and susceptibility to plasma proteases, FGF21 was difficult to use in clinical practice. Therefore, diverse chemical modifications have been used to increase its stability and applicability (112). Current clinical studies targeting FGF21 mainly focus on its ameliorative effects on metabolic diseases. The use of fibroblast activation protein inhibitors, BR103354, successfully elevates FGF21 levels in cynomolgus monkeys (113). Treatment of nonalcoholic fatty liver disease using bms-986036, a PEGylated FGF21 analog, resulting in a considerable decrease in liver fat and fibrosis in patients after 16 weeks of treatment, with very limited adverse effects (114).

Preclinical pharmacological studies have extensively validated the protective effects of FGF21 in damaged hearts. AMPK-FGF21 helped regulate the survival of cardiomyocytes under ischemic conditions after MI (56). FGF21 ameliorated atrial fibrillation and tachycardia by reducing excessive fibrosis (74). FGF21 has been shown to improve post-infarction arrhythmias and preserve electrophysiological function through mediating cardiac sodium current and inward rectifier potassium current (115). By improving FGFR1 phosphorylation, long-term FGF21 administration helped to alleviate high-fat-diet-induced left heart dysfunction and restored FGF21 sensitivity (116). In alcoholic cardiomyopathy, FGF21 improved adverse effects related to dysfunctional mitochondria by mediating autophagic pathways (117). However, a recent study reported that FGF21-FGFR4 signaling upregulated the activity of ERK1/2, enhancing diabetes mouse concentric cardiac hypertrophy and adverse cardiac remodeling (11). The FGF21 analog shows promise as a candidate for the diagnosis and therapy of cardiac diseases, but further investigation is needed to support its use in the future.

## Conclusion

The review mainly expounds on the possibility that FGF21, as a promising cardioprotective factor, improves cardiac function and ultimately retarding the progress of cardiac diseases. The actions of FGF21 for the heart are extensive. On the one hand, FGF21 has a regulatory effect targeted to different populations of cardiac tissue, including cardiomyocytes, immune cells, and fibroblasts. On the other hand, by acting on AMPK/SIRT1/PGC-1*α*, PI3K-Akt, ERK1/2, and TGF-*β*/Smad2 to mediate vital mechanisms, including autophagy, energy metabolism, oxidative stress, inflammation, and fibrosis, it is verified that FGF21 acts as a possible cardiac protective molecule. Therefore, the next steps in the development of relevant studies on targeted therapy of FGF21 in cardiac remodeling will be promising directions.
